# Collaborative care for the treatment of comorbid depression and coronary heart disease: a systematic review and meta-analysis protocol

**DOI:** 10.1186/2046-4053-3-127

**Published:** 2014-10-28

**Authors:** Phillip J Tully, Harald Baumeister

**Affiliations:** 1 Department of Rehabilitation Psychology and Psychotherapy, Institute of Psychology, University of Freiburg, Engelbergerstr. 41, Freiburg 79085, Germany; 2 Freemasons Foundation Centre for Men’s Health, Discipline of Medicine, School of Medicine, The University of Adelaide, Adelaide, Australia; 3 Medical Psychology and Medical Sociology, Faculty of Medicine, University of Freiburg, Freiburg, Germany

**Keywords:** Collaborative care, Systematic review, Meta-analysis, Randomized controlled trial, Cost-benefit analysis, Anti-depressant, Depression disorder, Major, Protocol

## Abstract

**Background:**

Depression and coronary heart disease (CHD) are frequently comorbid and portend higher morbidity, mortality and poorer quality of life. Prior systematic reviews of depression treatment randomized controlled trials (RCTs) in the population with CHD have not assessed the efficacy of collaborative care. This systematic review aims to bring together the contemporary research on the effectiveness of collaborative care interventions for depression in comorbid CHD populations.

**Methods/Design:**

Electronic databases (Cochrane Central Register of Controlled Trials MEDLINE, EMBASE, PsycINFO and CINAHL) will be searched using a sensitive search strategy exploding the topics CHD, depression and RCT. Full text inspection and bibliography searching will be conducted, and authors of included studies will be contacted to identify unpublished studies. Eligibility criteria are: population, depression comorbid with CHD; intervention, RCT of collaborative care defined as a coordinated model of care involving multidisciplinary health care providers, including: (a) primary physician and at least one other health professional (e.g. nurse, psychiatrist, psychologist), (b) a structured patient management plan that delivers either pharmacological or non-pharmacological intervention, (c) scheduled patient follow-up and (d) enhanced inter-professional communication between the multiprofessional team; comparison, either usual care, enhanced usual care, wait-list control group or no further treatment; and outcome, major adverse cardiac events (MACE), standardized measure of depression, anxiety, quality of life, cost-effectiveness. Screening, data extraction and risk of bias assessment will be undertaken by two reviewers with disagreements resolved through discussion. Meta-analytic methods will be used to synthesize the data collected relating to the outcomes.

**Discussion:**

This review will evaluate the effectiveness and cost-effectiveness of collaborative care for depression in populations primarily with CHD. The results will facilitate integration of evidence-based practice for this precarious population.

**Systematic review registration:**

PROSPERO CRD42014013653.

## Background

The prevalence of depression disorder is estimated at between 15%–20% in the coronary heart disease (CHD) population, while clinically relevant depressive symptoms are prevalent in up to 40% of this group [1-3]. Moreover, depression is widely documented to lead to an adverse CHD prognosis [4-8]. Specifically, depression disorder and depressive symptoms have been linked to a twofold higher risk of death [4] and a higher risk for subsequent major adverse cardiac events (MACE) including myocardial infarction or coronary revascularization [9]. Also, depression is associated with diminished quality of life [6,7,10] and failure to make gains in quality of life after coronary revascularization in longitudinal studies [11]. Considering that depression is a modifiable risk factor yet portends subsequent MACE risk, it has been hypothesized that depression intervention in the CHD population could lead to fortuitous benefits to an otherwise poorer CHD prognosis [12,13].

Unfortunately, prior psychological and pharmacological depression intervention efforts in the CHD population have reported only small, albeit significant, effects on depression symptoms [14]. Moreover, and conspicuously, previous trials including the landmark ENRICHD study [13] have not led to a significant reduction in MACE [14], raising questions about the design [15] and acceptability [16] of depression interventions in the population with CHD, especially in the period after an acute myocardial infarction. More recently, collaborative care has emerged as a potentially promising model of health care among populations with complex mental health needs [17] and also mental health needs comorbid with chronic diseases such as diabetes [18] and CHD [19]. Collaborative care is defined by a multi-professional approach to patient care delivered by a physician and at least one other health professional, involving a structured patient management plan and interventions, scheduled patient follow-ups and enhanced inter-professional communication between the multi-professional team [19].

In a 2012 review of collaborative care for depression in medically ill patients, it was noted that evidence was, at that time, sparse in relation to the efficacy of collaborative care and chronic disease-specific outcomes [20]. Moreover, the authors noted that there was no evidence for the cost-effectiveness of collaborative care in the population with chronic diseases [20], calling into question the applicability of this particular model of care to health care policy makers and clinicians. Subsequently, Huang and colleagues [18] reported a systematic review and meta-analysis of eight randomized controlled trials (RCTs) regarding collaborative care for depression comorbid with diabetes. The review revealed that collaborative care was associated with depression remission (risk ratio (RR) = 1.53, 95% confidence interval [CI] =1.11–2.12) indicating that evidence relating to the efficacy of collaborative care for chronic disease-specific populations is emerging [18].

However, the collaborative care evidence basis in the population with CHD has not been subject to systematic review and meta-analysis. Indeed, prior reviews [21-23] have evaluated a single RCT comprised of CHD patients [24], although mixed CHD and diabetes samples were included [19], whereas other collaborative care depression interventions in the CHD population [25] was included in only one review [23]. Further to such limitations to date, several large prospective RCTs of collaborative depression care have been reported in CHD populations [26-28] raising the possibility to evaluate the efficacy and early benefits of collaborative care. A systematic review of this type, as described herein in protocol stage, might in turn assist in the design of subsequent trials and inform clinicians. Moreover, an evaluation of the extant literature’s study quality and cost-effectiveness might inform clinical practice and assist health care policy makers’ integration of the results into models of care. Herein, we outline a systematic review and meta-analysis protocol designed to overcome the abovementioned limitations pertaining to collaborative care for depression in adults with comorbid depression and CHD.

## Methods and design

### Aims

The proposed review aims to synthesize the evidence base of RCTs reporting the effectiveness of collaborative depression care in persons with CHD with respect to MACE risk, depression, anxiety, quality of life and cost-effectiveness. The reporting of this review will conform to the PRISMA guidelines [29].

### Eligibility criteria

•Population: studies must be performed among adults (18 years and older) with comorbid depression and CHD. Depression must be defined as depression disorder or clinical depression assessed according to the Diagnostic and Statistical Manual of Mental Disorders (DSM) or International Classification of Diseases (ICD) by a standardized interview (e.g. Structured Clinical Interview, Composite International Diagnostic Interview). However, in favor of providing a more comprehensive overview, we will also include trials using validated self-reports or rating scales with specific cut-off points for depression. Mixed samples (e.g. heart failure, arrhythmia, diabetes) are eligible if ≥50% of the sample have a CHD diagnosis, as the first large collaborative care RCT for comorbid depression and chronic illness included persons with CHD or diabetes [19].

•Intervention: is a collaborative care intervention, with collaborative care defined as a coordinated model of care involving multidisciplinary health care providers, including: (a) primary physician and at least one other health professional (e.g. nurse, psychiatrist, psychologist); (b) a structured patient management plan that delivers either pharmacological or non-pharmacological intervention; (c) scheduled patient follow-up and (d) enhanced inter-professional communication between the multiprofessional team. Collaborative care may include usual CHD care or blended depression-CHD care.

•Comparison: a control group being either (enhanced) usual care, wait-list control group, or no further treatment for comorbid depression-CHD.

•Outcomes: primary, the primary outcome is all-cause and CHD-related mortality as well as non-fatal MACE (e.g. subsequent myocardial infarction, revascularization procedure, incident heart failure, stroke) and secondary, the secondary outcomes include the effects of collaborative care on depression (measured either dimensionally or categorically) following the intervention assessed by validated self-report questionnaires or standardized interview. Other secondary outcomes include anxiety and quality of life. Moreover, we will assess economic evaluations of health care costs or resource utilization and quality of life in collaborative care. Specifically, this includes cost cost-effectiveness (incremental cost-effectiveness ratio) and cost-utility (quality-adjusted life years).

#### Study type

Only RCTs that are peer-reviewed studies in full text, conference abstract or doctoral dissertations are eligible for this review. Studies must be published in English or German to be eligible. We will exclude cross-sectional studies, case series and case reports.

### Search strategy

We will identify relevant articles in any language by searching electronic databases from inception including: the Cochrane Central Register of Controlled Trials (CENTRAL) on The Cochrane Library, MEDLINE, EMBASE, PsycINFO and CINAHL. A highly sensitive RCT filter will be used with the MEDLINE search, as previously reported by Higgins [30]. Importantly, as not all interventions may identify as collaborative care (e.g. [27]), we will utilize a comprehensive and broad search used previously [14] encompassing the topics CHD, depression and RCT without being limited to specific interventions. The search strategy is provided in Additional file 1. We will hand search the reference lists of articles selected for full text to supplement the electronic searches. The principal investigators of studies will be contacted to clarify study eligibility if required. Primary authors will also be contacted to ascertain missing or unpublished data and their knowledge of any other collaborative care trials not retrieved by our primary search.

### Study selection process

Initially, two reviewers (PJT, HB) will independently screen titles and abstracts of all the retrieved bibliographic records. Full texts of all potentially eligible records passing the title and abstract screening level will be retrieved and examined independently by two reviewers with the abovementioned eligibility criteria [29]. Disagreements at both screening levels (title/abstract and full text) will be resolved through discussion or adjudication of a third reviewer. A PRISMA flow chart will outline the study selection process and reasons for exclusions.

### Data items for collection

After determination of the initial study, eligibility information will be extracted for each study pertaining to study identification (first author, year of publication, country where recruitment took place), study design characteristics (sample size, intervention design, duration of intervention, % receiving antidepressants, % receiving psychotherapy, number of intervention phone calls, length of follow-up) and patient population (age, gender, % CHD). Primary outcome data collected at the conclusion of the intervention will include MACE. Secondary outcome data will include depression severity, response and remission (measured either dimensionally or categorically), health-related quality of life, anxiety symptoms and cost-effectiveness. These variables will be extracted for all studies, after which the extracted data will be verified by a second reviewer to reduce reviewer errors and bias. All disagreements will be handled by consensus from the three adjudicators.

There is a likelihood of more than one reported eligible outcome per study [31], we will include data as follows: 1. in the case of available data from both rating scales (e.g. Hamilton Rating Scale for Depression) and self-report questionnaires (e.g. Beck Depression Inventory), data from rating scales will be prioritized and 2. in the case where several outcome measures of the same hierarchy level are used in one study, we will select the outcome measure that is used most frequently across the eligible studies.

### Risk of bias

Two reviewers will independently assess the risk of bias in duplicate of the included studies using the Cochrane Collaboration’s tool for assessing risk of bias in RCTs [30]. With regard to psychological interventions, blinding of health care providers or patients concerning the treatment is not feasible, but an evaluation of outcomes can be performed by researchers that are unaware of the treatment allocation of patients. Trials of psychological interventions will therefore be evaluated regarding the blinding of the outcome assessors. In pharmacological trials, blinding is possible for patients, personnel and outcome assessor and will be evaluated accordingly. The impact of possible bias in altering the results will then be evaluated, and conclusions concerning an overall risk of bias for primary and secondary outcomes will be drawn. For each included RCT, we will provide a description, comment and judgment of risk as ‘Low’ , ‘Unclear’ or ‘High’ , for each of the items: random sequence generation, allocation concealment, blinding of participants and personnel (subjective and objective outcomes), incomplete outcome data, selective reporting and other bias (see Table 1).

**Table 1:** Outline of risk of bias assessment

**Aspect of trial bias assessed**	**Type of risk of bias in trial and clarification**
1. Random sequence generation	Selection bias
2. Allocation concealment	Selection bias
3. Blinding of participants and personnel	Performance bias
4a. Blinding of outcome assessment - subjective outcomes	Detection bias; e.g. depression symptoms measured on a standardized self-report questionnaire
4b. Blinding of outcome assessment - objective outcomes	Detection bias; e.g. depression remission measured by a structured clinical interview
5. Incomplete outcome data	Attrition bias
6. Selective reporting	Reporting bias
7. Other bias	e.g. when bias may seriously alter the results, such as improper adjustment for baseline imbalances or funding bias

### Synthesis of data and summary measures

#### Data synthesis and meta-analysis

For all included RCTs, we will provide a detailed description of the results in both tables and text. We will use RevMan 5.2 to conduct the meta-analyses. When data is available to be pooled together, we will use a random-effects model using the inverse-variance method outlined by DerSimonian and Laird which is a more conservative estimate of effect size [32]. Where possible, we will aggregate each included RCT’s MACE data for the intervention and control group as dichotomous variables with the RR and 95% CI. When studies report depression and/or anxiety remission among the intervention and control group as a dichotomous outcome, we will also aggregate this data with the RR and 95% CI. Health care costs reported as incremental cost-effectiveness ratio will be aggregated with the RR and 95% CI. Otherwise, continuous outcome data (e.g. depression symptoms, anxiety symptoms, quality of life, total costs) between the intervention and control group will be analyzed with the SMD. The SMD is calculated as the difference between the intervention and control means in each trial divided by the estimated between-person standard deviation (SD) for that trial.

Due to the nature of outcomes assessed in the review, it is possible that studies will report multiple observations with heterogeneity concerning the follow-up length of the outcome assessment [14,31]. We will analyse follow-up durations using different time frames: 1. short term (up to 6 months post-treatment); 2. medium term (6–12 months post-treatment) and 3. long term (more than 12 months post-treatment). Our inclusion criteria do not specify requisite time points at which outcomes are measured to avoid excluding potentially useful information; however, we will account for timing during data analysis. We intend to group all studies together initially and then perform sensitivity analyses for different time points (e.g. depression in the short, medium and long term), if possible.

It is possible that individual studies may consist of multiple treatment groups, such as different types of depression interventions or different doses of medication. In order to avoid the possibility of introducing bias caused by multiple statistical comparisons with one control group, we will combine the groups from multiple-arm studies into a single group.

### Assessment of heterogeneity

Heterogeneity will be evaluated with the *I*^2^ statistic. According to the Cochrane Handbook for Systematic Reviews of Interventions [30], *I*^2^ of 0%–60% can be regarded as not important to moderate (0%–60%), while *I*^2^ > 60% indicates substantial heterogeneity. The results of this assessment will guide the decision whether to perform meta-analyses or not and whether to base the analyses on a random-effects or a fixed-effect model.

We have considered the following to explain potential sources of heterogeneity between the included RCT studies: (1) trials with a higher risk of bias will show larger effects than trials with lower risk of bias [30] and (2) trials comprised by a proportion of patients with diabetes will contain larger effect sizes than trials consisting of CHD patients only [14,33].

### Assessment of publication bias

The presence of publication bias will be evaluated using the test of Egger et al. [34] and the funnel plot.

### Planned subgroup analyses and sensitivity analyses

Sensitivity analyses will assess sub-groups restricted to (1) studies consisting of only CHD patients and not inclusive of mixed chronic diseases such as diabetes, (2) studies utilizing a standardized psychiatric interview and (3) studies with a low overall risk of bias.

### GRADE framework

The proposed review will use the Grading of Recommendations Assessment, Development and Evaluation (GRADE) guidelines [35] to determine the quality and strength of recommendations.

## Discussion

This systematic review may add to the extant literature by reporting the efficacy of collaborative care depression interventions in CHD populations with respect to MACE, depression, anxiety, quality of life and health care costs. It is feasible that the findings of our review will extend beyond previous systematic reviews including a Cochrane review in the coronary artery disease population [14]. As such, the proposed systematic review will be interpreted alongside prior high-quality systematic reviews of depression treatment in CHD [14] and those prior systematic reviews pertaining to collaborative depression care in diabetes [18] and chronic illnesses [22,23]. The findings might therefore serve to inform the design of future RCTs, evidence-based clinical practice and health care policy, especially relating to the effectiveness and cost-effectiveness of collaborative depression care in CHD.

There are several limitations that will contextualize the findings and generalizability of the proposed review including the relative infancy of the collaborative care literature in the CHD population with depressive symptoms. Limitations of the original studies may also include between-study heterogeneity and high risk of bias that will potentially limit the conclusions drawn. Specifically, although blinding of treatment allocation is not feasible in depression RCTs, it is possible that systematic bias may relate to assessment of depressive symptoms by non-blinded study assessors. Moreover, trials may utilize different depression measures, incorporating different symptoms with discrepant validity in the population with CHD. Therefore, the proposed review may be limited by the pooling together of depression and other patient-reported outcome measures with divergent psychometric properties. Finally, despite attempts to retrieve unpublished and non-significant studies, the proposed systematic review is likely to be limited by publication bias of only significant findings, given the infancy of the literature [36]. Finally, as the proposed review will include only English and German language studies, the generalizability of the findings from studies published in other languages and other health care settings is limited.

In conclusion, given the emergence of collaborative care depression intervention evidence in chronic disease populations including CHD, and the absence of a systematic review, this review will help in summarizing the available evidence, both quantitatively and qualitatively.

## Abbreviations

CHD: Coronary heart disease; CI: Confidence interval; MACE: Major adverse cardiac events; OR: Odds ratio; RCT: Randomized controlled trial; SMD: Standardized mean difference.

## Competing interests

The article processing charge was funded by the Open Access publication fund of the Albert Ludwigs University of Freiburg. PJT is supported by the National Health and Medical Research Council of Australia (Neil Hamilton Fairley - Clinical Overseas Fellowship #1053578). The funders had no role in study design, data collection and analysis, decision to publish or preparation of the manuscript. The authors declare that they have no competing interests.

## Authors’ contributions

PJT and HB were involved in the concept and review design of the study and data analysis plan. Both authors had major contributions to the write-up and editing of the manuscript. Both authors read and approved the final manuscript.

## Supplementary Material

Additional file 1 Search strategy. Click here for file
