# Supplementary material for: Collaborative care for the treatment of comorbid depression and coronary heart disease: a systematic review and meta-analysis protocol
Source: Syst Rev. 2014 Oct 28;3:127. doi: 10.1186/2046-4053-3-127 (PMC4214823; doi:10.1186/2046-4053-3-127)
Supplement: Additional file 1 — Search strategy. [file 2046-4053-3-127-S1.pdf]

Appendix 1

Search strategy

CENTRAL, DARE, HTA and EED on The Cochrane Library

#1 MeSH descriptor myocardial ischemia explode all trees

#2 MeSH descriptor Myocardial Revascularization explode all trees

#3 (ischemi* in All Text near/3 heart in All Text)

#4 (ischaemi* in All Text near/3 heart in All Text)

#5 (coronary in All Text near/3 disease* in All Text)

#6 angina in All Text

#7 myocardial next infarct* in All Text

#8 heart next infarct* in All Text

#9 (coronary in All Text near/3 bypass in All Text)

#10 (heart in All Text near/3 disease in All Text)

#11 (cardiac in All Text near/3 disease in All Text)

#12 chd in All Text

#13 cad in All Text

#14 (coronary in All Text near/3 angioplasty in All Text)

#15 (#1 or #2 or #3 or #4 or #5 or #6 or #7 or #8 or #9 or #10)

#16 (#11 or #12 or #13 or #14)

#17 (#15 or #16)

#18 MeSH descriptor depression explode all trees

#19 MeSH descriptor Depressive Disorder explode all trees

#20 MeSH descriptor Mood Disorders this term only

#21 “depression” in Keywords

#22 “depressive” in Keywords

#23 “Dysthymia” in Keywords

#24 dysthymi* in All Text

#25 (depressi* in All Text near/3 disorder* in All Text)

#26 (depressi* in All Text near/3 symptom* in All Text)

#27 mood next disorder* in All Text

#28 depression in Record Title

#29 antidepress* in All Text

#30 anti-depress* in All Text

#31 (#18 or #19 or #20 or #21 or #22 or #23 or #24 or #25 or #26 or #27)

#32 (#28 or #29 or #30)

#33 (#31 or #32)

#34 (#17 and #33)

Search strategy MEDLINE (on Ovid)

1 exp Myocardial Ischemia/

2 exp Myocardial Revascularization/

3 (isch?emi$ adj3 heart).tw.

4 (coronary adj3 disease).tw.

5 angina.tw.

6 myocardial infarct$.tw.

7 heart infarct$.tw.

8 (coronary adj3 bypass$).tw.

9 (heart adj3 disease).tw.

10 (cardiac adj3 disease).tw.

11 chd.tw.

12 CAD.tw.

13 (coronary adj3 angioplasty).tw.

14 or/1-13

15 Depression/

16 exp Depressive Disorder/

17 Mood Disorders/

18 dysthymi$.tw.

19 (depressi$ adj3 disorder$).tw.

20 (depressi$ adj3 symptom$).tw.

21 mood disorder$.tw.

22 affective disorder$.tw.

23 antidepress$.tw.

24 anti-depress$.tw.

25 or/15-24

26 14 and 25

27 randomized controlled trial.pt.

28 controlled clinical trial.pt.

29 randomized.ab.

30 placebo.ab.

31 drug therapy.fs.

32 randomly.ab

33 trial.ab.

34 groups.ab.

35 or/27-34

36 humans.sh.

37 35 and 36

38 37 and 26

EMBASE (OVID)

1 exp ischemic heart disease/

2 exp coronary artery surgery/

3 exp percutaneous coronary intervention/

4 (isch?emi$ adj3 heart).tw.

5 (coronary adj3 disease).tw.

6 angina.tw.

7 myocardial infarct$.tw.

8 heart infarct$.tw.

9 (coronary adj3 bypass$).tw.

10 (heart adj3 disease).tw.

11 (cardiac adj3 disease).tw.

12 chd.tw.

13 CAD.tw.

14 (coronary adj3 angioplasty).tw.

15 or/1-14

16 exp depression/

17 affective neurosis/

18 Mood Disorder/

19 dysthymi$.tw.

20 (depressi$ adj3 disorder$).tw.

21 (depressi$ adj3 symptom$).tw.

22 mood disorder$.tw.

23 affective disorder$.tw.

24 antidepress$.tw.

25 anti-depress$.tw.

26 or/16-25

27 15 and 26

28 controlled clinical trial/

29 random$.tw.

30 randomized controlled trial/

31 follow-up.tw.

32 double blind procedure/

33 placebo$.tw.

34 placebo/

35 factorial$.ti,ab.

36 (crossover$ or cross-over$).ti,ab.

37 (double$ adj blind$).ti,ab.

38 (singl$ adj blind$).ti,ab.

39 assign$.ti,ab.

40 allocat$.ti,ab.

41 volunteer$.ti,ab.

42 Crossover Procedure/

43 Single Blind Procedure/

44 or/28-43

45 (exp animals/ or nonhuman/) not human/

46 44 not 45

47 27 and 46

PsycINFO

1 exp heart disorders/

2 heart surgery/

3 (isch?emi$ adj3 heart).tw.

4 (coronary adj3 disease).tw.

5 angina.tw.

6 myocardial infarct$.tw.

7 heart infarct$.tw.

8 (coronary adj3 bypass$).tw.

9 (heart adj3 disease).tw.

10 (cardiac adj3 disease).tw.

11 chd.tw.

12 CAD.tw.

13 (coronary adj3 angioplasty).tw.

14 or/1-13

15 exp affective disorders/

16 “depression (emotion)”/

17 dysthymi$.tw.

18 (depressi$ adj3 disorder$).tw.

19 (depressi$ adj3 symptom$).tw.

20 mood disorder$.tw.

21 affective disorder$.tw.

22 antidepress$.tw.

23 anti-depress$.tw.

24 or/15-23

25 14 and 24

26 random$.tw.

27 ((singl$ or doubl$ or trebl$ or tripl$) adj25 (blind$ or dummy or mask$)).tw.

28 placebo$.tw.

29 crossover.tw.

30 assign$.tw.

31 allocat$.tw.

32 ((clin$ or control$ or compar$ or evaluat$ or prospectiv$) adj25 (trial$ or studi$ or study)).tw.

33 placebo/

34 treatment effectiveness evaluation/

35 mental health program evaluation/

36 experimental design/

37 clinical trials/

38 or/26-37

39 25 and 38

CINAHL (EBSCO)

((MH “Affective Disorders+”) or (TI depression) or dysthymi* or (mood disorder*) or (affective disorder*) or antidepress* or antidepress* or (depressi* N3 disorder*) or (depressi* N3 symptom*)) and ((MH “Myocardial Ischemia+”) or (MH “Myocardial Revascularization+”) or Angina or (myocardial infarct*) or (heart infarct*) or coronary or cardiac or chd or CAD or (heart disease)) and ((MH “Clinical Trials+”) or randomi* or randomly or placebo* or trial)
